# Some Physical Properties of Protein Moiety of Alkali-Extracted Tea Polysaccharide Conjugates Were Shielded by Its Polysaccharide

**DOI:** 10.3390/molecules22060914

**Published:** 2017-05-31

**Authors:** Xiaoqiang Chen, Wei Song, Jin Zhao, Zhifa Zhang, Yuntian Zhang

**Affiliations:** 1 College of Bioengineering and Food, Hubei University of Technology, Wuhan 430068, China; jinan1616@163.com; 2 Department of Food Science and Engineering, MIITKey Laboratory of Critical Materials Technology for New Energy Conversion and Storage, School of Chemistry and Chemical Engineering, Harbin Institute of Technology, Harbin 150090, China; poly2002@163.com; 3 College of Life Science, China Jiliang University, Hangzhou 310018, China; herb_cas@163.com; 4 Tongji Medical College, Huazhong University of Science and Technology, Wuhan 430030, China; lituo580@163.com

**Keywords:** alkali-extracted, tea polysaccharide conjugates, protein

## Abstract

Polysaccharide conjugates were alkali-extracted from green tea (TPC-A). Although it contained 11.80% covalently binding proteins, TPC-A could not bind to the Coomassie Brilliant Blue dyes G250 and R250. TPC-A had no expected characteristic absorption peak of protein in the UV-vis spectrum scanning in the range of 200–700 nm. The UV-vis wavelength of 280 nm was not suitable to detect the presence of the protein portion of TPC-A. The zeta potential of TPC-A merely presented the negative charge properties of polysaccharides instead of the acid–base property of its protein section across the entire pH range. Furthermore, TPC-A was more stable when the pH of solution exceeded 4.0. In addition, no precipitation or haze was generated in the TPC-A/(−)-epigallocatechin gallate (EGCG) mixtures during 12 h storage. TPC-A has emulsifying activity, which indicated that its protein moiety formed hydrophobic groups. Thus, it was proposed that some physical properties of TPC-A protein were shielded by its polysaccharide, since the protein moiety was wrapped by its polysaccharide chains.

## 1. Introduction

Tea polysaccharide conjugates (TPC)—one of the main functional components in tea—are protein-bound acidic polysaccharide conjugates. Tea polysaccharide conjugates have gained increasing attention in recent years because of investigation of their hypoglycemic functions, immunoregulation, and antioxidant activity [1,2,3,4,5,6]. Most of all, many results demonstrated that tea polysaccharide conjugates have great potential against diabetes and will be a promising supplement for promoting human health. At present, researches including the purification, physicochemical properties, and bioactivities have mainly focused on hot water-extracted tea polysaccharide conjugates [3]. However, alkali-extracted tea polysaccharide conjugates (TPC-A) are more plentiful in tea, but have rarely been studied—especially their protein moiety. Our previous study has shown that the residue—the by-product remaining from tea after water extraction—can also be further extracted with dilute alkali to obtain a large number of polysaccharide substances that likewise have hypoglycemic effect [2]. Thus, further studies of the physicochemical properties of TPC-A can make full use of low-grade tea and by-product from tea drinks manufacture, and meanwhile accelerate the development of TPC application. As is known, Coomassie Brilliant Blue dye G-250 is generally used in SDS-PAGE for the detection of protein, and the R250 dye can be applied to the Bradford method for protein content determination. There are characteristic absorption peaks, especially at about 280 nm in the UV-vis spectrum of protein. EGCG can combine with protein to form a precipitate. Protein possesses acid–base ionization in a certain pH range, and zeta potential shows negative and positive values in solution pH greater or less than their isoelectric point. The amphipathic property of protein usually gives it emulsifying properties. To date, whether the protein in the polysaccharide conjugate also has emulsifying properties is not yet known.

This research investigated the physical properties of TPC-A protein moiety by evaluating its ability to bind to the Coomassie Brilliant Blue dyes G250 and R250, zeta potential as a function of pH, ultraviolet characteristic absorption , stability of TPC-A and (−)-epigallocatechin gallate (EGCG) mixtures, emulsifying property, and hygroscopicity. This research may provide the technical parameters of the quality control and approaches for the further application of TPC-A.

## 2. Results and Discussion

### 2.1. Coomassie Brilliant Blue Dye Detection of the Protein Portion of TPC-A

Amino acid analysis showed that the protein portion of TPC-A consisted of 16 amino acids (mg·g^−1^): Asp 18.49, Ser 7.65, Glu 20.45, Gly 11.30, Arg 8.17, Thr 6.75, Ala 9.23, Pro 7.86, Val 9.96, Lys 6.43, Ile 7.40, Leu 11.49, Phe 7.26, His 0.27, Tyr 4.22, Met 0.07 [2]. According to the amino acid composition, the overall content of amino acid in TPC-A was 13.73% (*w*/*w*), while free amino acids were not detected. The free amino acids and other small molecules in tea were removed by ethanol precipitation and dialysis during TPC-A preparation.

According to the overall amino acid content, the content of protein in TPC-A is calculated as the following formula: 13.73% × 110/128 = 11.80% (*w*/*w*).

**Figure 1:**
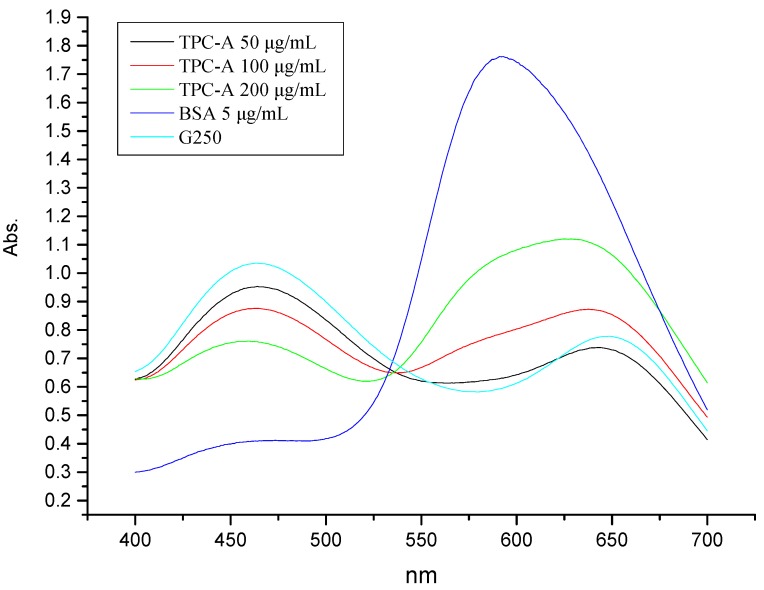
UV-vis absorption spectra of alkali-extracted tea polysaccharide conjugates (TPC-A) and bovine serum albumin (BSA) in Coomassie Brilliant Blue G250 solution.

The absorption spectra of TPC-A and Coomassie Brilliant Blue dye G250 mixture are shown in Figure 1. There was only one absorption peak appearing at 594 nm in the scanning spectrum of 5 µg/mL bovine serum albumin (BSA) in Coomassie Brilliant Blue G250 solution, which abided by the principle of Bradford method for protein assay. According to the principle, the maximum absorption is at about 595 nm when the G250 dye is bound to protein [7]. According to the protein content of TPC-A (11.80%), 50, 100, and 200 µg/mL of TPC-A dissolved in Coomassie Brilliant Blue dye G250 solution were equivalent to 5.90, 11.80, and 23.60 µg/mL of protein, respectively. From Figure 1, two characteristic absorption peaks emerged at 645 nm and 465 nm in the three scanning spectra of TPC-A in G250 dye solution, which were the same as that of the Coomassie Brilliant Blue G250 solution. This result indicated that TPC-A did not bind to Coomassie Brilliant Blue dye G250, although it contained protein. In addition, no TPC-A bands were detected in SDS-PAGE, which also indicated that Coomassie Brilliant Blue dye R250 did not bind to the protein portion of TPC-A. GC-MS analysis indicated that TPC-A was composed of seven monosaccharides, including rhamnose, fucose, arabinose, xylose, mannose, glucose, and galactose, with molar ratios of 13.81:1.43:36.07:5.24:4.89:6.28:32.27.

Coomassie Brilliant Blue G250 and R250 were unable to detect protein in TPC-A. It is proposed that the protein of TPC-A is wrapped by its polysaccharide chains, which prevent the TPC-A protein from binding to Coomassie Brilliant Blue dye.

On the other hand, the above results demonstrate that Bradford method does not apply to the determination of the protein content of TPC and even other plant polysaccharide conjugates.

### 2.2. UV-Vis Spectrum Analysis of TPC-A Aqueous Solution

**Figure 2:**
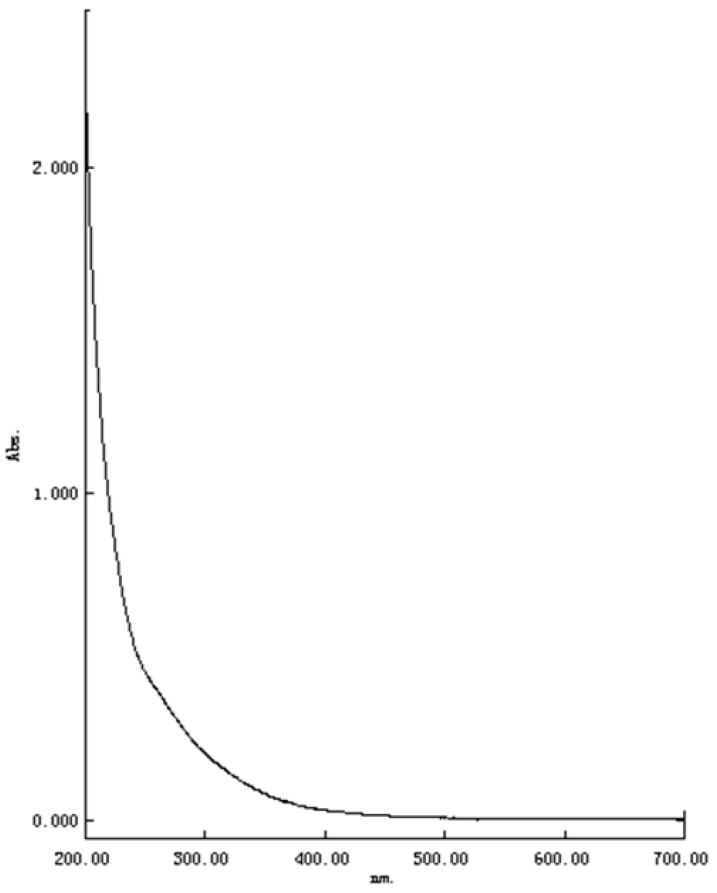
UV-vis spectrum of TPC-A aqueous solution (100 μg/mL) scanned in the wavelength range of 200–700 nm.

As shown in Figure 2, TPC-A has no characteristic absorption peak in the UV-vis spectrum scanning in the range of 200–700 nm. Although TPC-A contains more than 10% protein, its spectrum did not produce the expected ultraviolet absorption characteristics of protein.

Three kinds of amino acids—tryptophan (Trp), tyrosine (Tyr), and phenylalanine (Phe)—have characteristic ultraviolet absorption [8]. The absorption maximums of Trp, Tyr, and Phe are at 280 nm, 275 nm, and 257 nm, with the molar extinction coefficients ε 280 = 5600, ε 275 = 1400, and ε 257 = 200, respectively [8]. The amino acid compositions demonstrated that TPC-A contained Tyr and Phe residue (except Trp residue owing to its hydrolysis in the amino acid measurement even if it exists). However, the characteristic adsorptions of Tyr and Phe were not observed in the UV-vis spectrum of TPC-A. This phenomenon further verified the proposed speculation that the protein of TPC-A was wrapped by its polysaccharide chains and some physicochemical properties of the protein moiety in TPC-A such as characteristic absorption peaks in the UV-vis spectrum are shielded.

In many previous studies, 280 nm has been used as the protein monitoring wavelength, especially for purification by column chromatography and determination, since tea polysaccharide conjugates contain protein [9,10]. However, our research indicates that the 280 nm UV-vis wavelength is not suitable for the detection of the protein moiety in TPC-A due to the lack of characteristic absorption peaks in the UV-vis spectrum scanning from 200 nm to700 nm.

### 2.3. Zeta Potentials and Stability of TPC-A Aqueous Solution as a Function of pH

**Figure 3:**
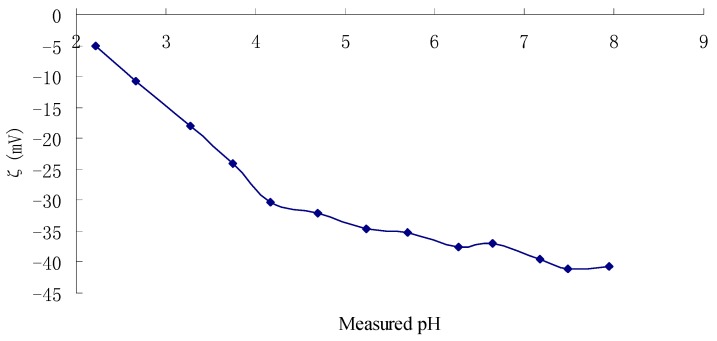
Zeta potentials of TPC-A as a function of pH 2.0–8.0 at an interval of 0.5.

Figure 3 shows zeta potentials of TPC-A at pH 2.0–8.0. It can be seen that all the zeta potential values of TPC-A are negative in both acidic and basic solutions, which demonstrates that TPC-A is an acidic polysaccharide conjugate. Proteins possess different isoelectric points due to the acid–base ionization. However, the zeta potentials curve of TPC-A exhibited no isoelectric point—a particular value of solution pH at which the net charge on the surface of the molecules or particles is zero. TPC-A aqueous solution merely presented the negative charge properties of its polysaccharides instead of the acid–base property of its protein section. It was interpreted as the protein portion of TPC-A being shielded by polysaccharide chains.

In addition, zeta potential is the key indicator for the measurement of the strength of attraction and repulsive force among large molecules or particulates in solution, which can be used to evaluate the solution stability.

The higher the absolute value of zeta potential (positive or negative), the greater the stability of the solution system. From Figure 3, the negative surface charges of TPC-A increased in the solution with the increasing of pH, and as a result, the zeta potential increased from −5.17 to −41.10 mV with increasing pH of the solution from 2.0 to 8.0. Zeta potential of TPC-A reached −30.33 mV at pH 4.17. The zeta potential curve indicated that TPC-A was more stable in basic solution. It is proposed that the pH of liquid product containing TPC-A should be greater than 4.0.

### 2.4. Stability of TPC-A and EGCG Aqueous Mixtures

**Figure 4:**
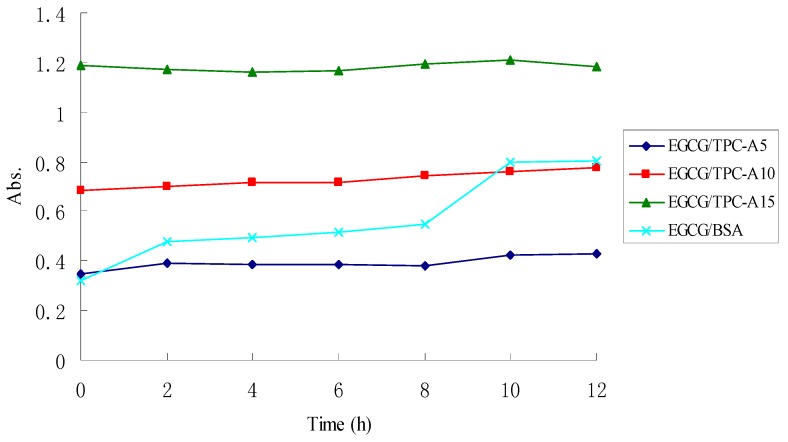
The turbidity evolution curve of TPC-A/EGCG aqueous mixtures at 520 nm during 12 h storage.

Figure 4 shows the turbidity changes of TPC-A and EGCG aqueous mixtures detected at 520 nm during their storage. The aqueous mixtures of BSA and EGCG were used as control. BSA/EGCG aqueous mixtures did not produce any macroscopic precipitation or haze in the beginning until the mixture was stored for 2 h. From Figure 4, an inflection point appeared at 2 and 8 h in the turbidity evolution curve of BSA/EGCG aqueous mixtures. However, no precipitation or haze occurred in the three TPC-A/EGCG aqueous mixtures through macroscopic observation during 12 h storage. In addition, there was no significant difference in the turbidity of the TPC-A/EGCG aqueous mixtures during the 12 h storage (Figure 4). The turbidity evolution curves of TPC-A/EGCG or BSA/EGCG aqueous mixtures are in accordance with their macroscopic observations.

Interaction force between polyphenols and protein are hydrogen bonding or hydrophobic bond [11,12,13]. Proteins containing proline residues are prone to produce precipitate when combined with polyphenols [11]. The result of amino acids composition showed that TPC-A contained proline residues. In this study, 5, 10, and 15 mg/mL TCP-A solutions, which contained 0.59, 1.18, and 2.36 mg/mL protein, respectively, were mixed with 8.0 mg/mL EGCG solution. From Figure 4, 8.0 mg/mL EGCG could form precipitate with 1.0 mg/mL BSA, but could not form precipitate with three concentrations of TPC-A. These results suggested that the protein portion of TPC-A could not undergo complexation with EGCG. This is further proof that TPC-A polysaccharide chains serve as a “shield” to screen its protein portion.

### 2.5. Stability of Medium-Chain Triglyceride (MCT) Emulsion Stabilized with TPC-A

**Figure 5:**
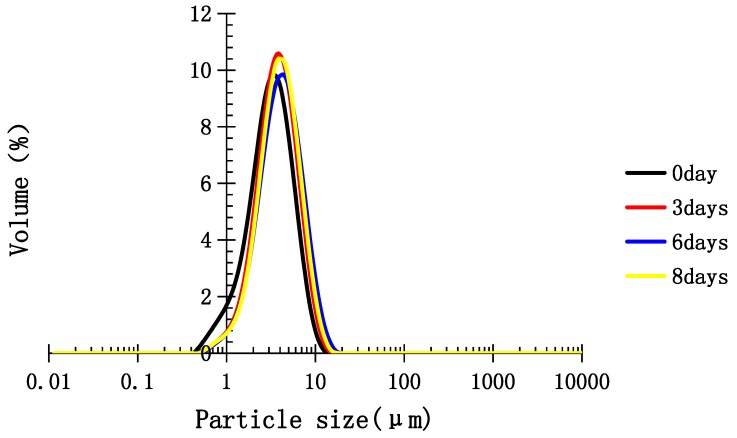
Particle size distribution (PSD) of medium-chain triglyceride (MCT) emulsion stabilized with TPC-A in the storage test at 25 °C for a period of 8 days.

**Figure 6:**
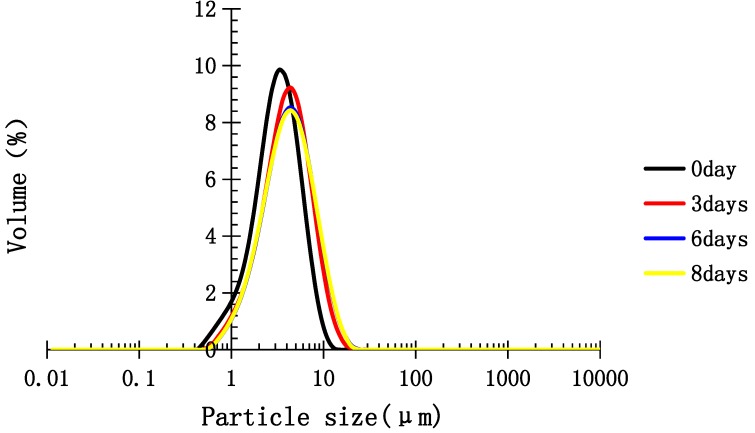
Particle size distribution (PSD) of MCT emulsion stabilized with TPC-A in the storage acceleration test at 60 °C for a period of 8 days.

From Figure 5, the particle size distribution (PSD) of MCT emulsion stabilized with TPC-A showed a single peak in the range of 1–10 μm, with an initial average particle size of 3.304 μm. The average particle size of the emulsion stored at 25 °C increased from 3.858 μm on day 3 to 4.071 μm on day 8, and increased from 4.256 μm on day 3 to 4.566 μm on day 8 at 60 °C. The PSD of MCT emulsion stabilized with TPC-A showed a single peak in the range of 1–10 μm (Figure 5 and Figure 6). MCT emulsion containing no TPC-A appeared stratified on day 3.

Some biomacromolecules possessing hydrophilic and hydrophobic groups have emulsifying activity [14]. In terms of the polysaccharide–protein conjugate, the large size and hydrophilicity of the polysaccharide moiety generates long-range steric repulsion between emulsion droplet surfaces, and the hydrophobic groups of the protein firmly anchor the conjugate to the oil–water interface [14].

The emulsifying capacity of TPC-A precisely indicates that its protein moiety forms hydrophobic groups. The hydrophobic groups reduce the sensitivity of TPC-A responding to the Coomassie Brilliant Blue G250 and R250, EGCG, and UV absorption, and lead to the negative charge of the zeta potential in aqueous solution. This newfound result with respect to the emulsifying capacity of TPC-A likewise verified that its protein portion was shielded by polysaccharide chains. The emulsifying capacity of TPC-A as well as its fine stability with EGCG have important theoretical value in the product development field, such as tea drinks containing TPC-A or an emulsifier, and simultaneously reveal the nature of its protein moiety.

### 2.6. Water Vapor Sorption Properties

**Figure 7:**
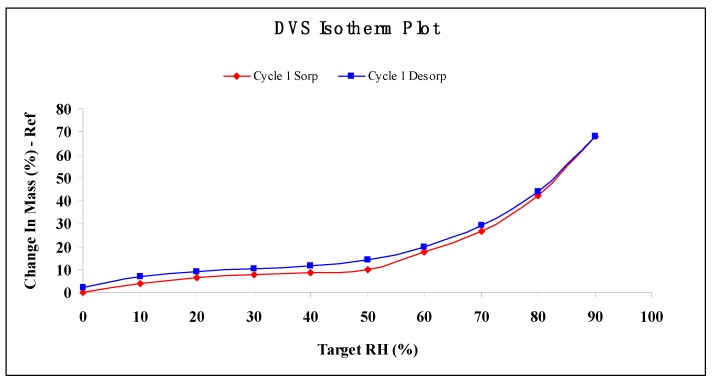
Water sorption/desorption properties of TPC-A. DVS: dynamic vapor sorption; RH: relative humidity.

Figure 7 clearly shows that the moisture sorption–desorption isotherm of TPC-A is a typical S-shaped curve. From 0% to 50% relative humidity (RH) in water vapor, TPC-A absorbed no more than 10% of its dry weight (9.71%). From 60% to 90% RH, the moisture sorption capacity of TPC-A grew quickly, increasing from 17.79% to 68.09% of its dry weight. The sorption–desorption isotherms of TPC-A exhibit hysteresis, and the amount of water associated with the solid is greater for the desorption isotherm than for the sorption isotherm between 0% and 80% RH. This curve showed that TPC-A had strong water retention under high humidity conditions greater than 80% RH.

Dynamic vapor sorption results showed that the moisture adsorption capability of TPC-A was the same as polysaccharide conjugates prepared from fresh tea leaves [15], and surpassed those of microcrystalline cellulose and chitosan as previously reported [16,17]. It is suggested that the humidity condition for keeping TPC-A in long-term preservation is below 60% RH.

## 3. Materials and Methods

### 3.1. Materials and Reagents

Low-grade green tea was purchased from Xihu County, Hangzhou city, China. Bovine serum albumin (BSA) was purchased from Sigma Co. (St. Louis, MO, USA). EGCG (96.82%) was purchased from Jiangxi Lvkang Co. (Nanchang, China). Coomassie Brilliant Blue R-250 and G-250 Dyes were purchased from Thermo Fisher Scientific Inc. (Shanghai, China). The standard monosaccharides (l-rhamnose, d-galactose, d-fucose, d-glucose, d-mannose, d-xylose, d-arabinose, d-glucuronic acid, and d-galacturonic acid) were purchased from Merck Co. (Darmstadt, Germany). Triethylamine and acetonitrile for chromatography were of HPLC grade from Tedia (Fairfield, CA, USA). The AccQ-Tag Ultra Chemistry Package was obtained from Waters (Milford, MA, USA). All other reagents were of analytical grade unless otherwise specified.

### 3.2. Polysaccharide Conjugates Prepared from Green Tea through Alkaline Extraction Method

Low-grade green tea (250 g) was ground, suspended in 3500 mL of distilled water, and incubated at 90 °C for up to 2 h under continuous stirring condition. The residue was used for the extraction by hot alkali. The residue was subsequently incubated at 50 °C for 2 h with 4 L of 1.0% aqueous sodium hydroxide. The extract from the residue was filtered, concentrated, adjusted to pH 9.0 by acetic acid, bleached with H_2_O_2_, precipitated, dialyzed, and lyophilized as described above to obtain alkali-extracted tea polysaccharides conjugates, termed TPC-A [2].

### 3.3. Monosaccharide Compositions

The monosaccharide composition of the polysaccharide moiety of TPC-A was determined through gas chromatography method. Samples in which myoinositol was added as the internal standard were hydrolysed in 2 M trifluoroacetic acid (TFA) at 120 °C for 2 h. The hydrolysate was converted into the alditol acetates via facile acetylation with pyridine and acetic anhydride (1:1, v/v) following reduction with NaBH4. Subsequent analysis was performed on a Hewlett-Packard HP 6890 gas chromatogram (Agilent Technologies Inc., Santa Clara, CA, USA) equipped with a fused-silica capillary column DB-225 (60 m × 0.25 mm, id; film thickness, 0.25 μM) and a flame-ionization detector (FID). The injector and detector temperatures were 250 and 270 °C, respectively. A temperature program of 190 °C (3 min) and 4 °C min−1 to 230 °C was used with helium as a carrier gas (1.2 mL/min flow rate). Prior to the detection of samples, the standard monosaccharides (l-rhamnose, d-galactose, d-fucose, d-glucose, d-mannose, d-xylose, and d-arabinose) and myoinositol were assessed for the identification of their respective typical retention times and electron impact profiles according to the identical procedure.

### 3.4. Amino Acid Compositions Protein Content

In the case of amino acid constituents (except for tryptophan (i.e., Trp), due to its inevitable degradation in the hydrolyzation reaction), solutions of the samples in 6 M HCl were incubated at 110 °C for 2 h. Subsequent pre-column derivatization of the resulting hydrolysates was performed with 6-aminoquinolyl-nhydroxysuccinimidyl carbamate (AQC). An Alliance system (Waters Corp., Milford, MA, USA) equipped with 2695 Separation Module, 2996 Photodiode Array Detector, and 2475 Multi λ Fluorescence Detector was used for HPLC analysis, as described previously [9]. For the separation of compounds, an AccQ-Tag reversed-phase dC18 column (4 μm, 3.9 mm × 150 mm) thermostated at 37 °C was used, and the eluted AQC derivatives were detected by monitoring their fluorescence (λex = 250 nm, λem = 395 nm).

Residue quantity for free amino acids (RQFAA) in TPC-A was determined through ninhydrin method [8].

The protein contents were determined according to the amino acids in TPC-A. The amino acids composition of TPC-A was detected. The overall content of amino acids (OCAA) in TPC-A was summarized.

There are about 20 amino acids synthesizing proteins in organisms, and the average molecular weight of these amino acids is 128 Da [8]. Accordingly, that of the amino acid residues in protein is 110 Da due to dehydration condensation reaction.

The content of protein in TPC-A is calculated as the following formula: (OCAA − RQFAA) × 110/128.

### 3.5. Coomassie Brilliant Blue Dye Detection of Protein in TPC-A

A 2 mg/mL TPC-A aqueous solution was prepared before use. Coomassie Brilliant Blue dye G250 solution was prepared as described by Bradford [7]. TPC-A/Coomassie Brilliant Blue dye G250 mixtures were determined by spectrum scanning, which was recorded in the wavelength range of 400–700 nm using a TU-1990 UV spectrophotometer (Persee General Instrument Co., Beijing, China). Three concentrations of TPC-A (50, 100, and 200 µg/mL) and 5 µg/mL BSA testing solutions were prepared using Coomassie Brilliant Blue dye G250. Coomassie Brilliant Blue dye G250 solution was also scanned in the wavelength range of 400–700 nm using a TU-1990 UV spectrophotometer (Persee General Instrument Co., Beijing, China) in the same way.

SDS-PAGE was employed to test TPC-A binding with Coomassie Brilliant Blue dye R250. SDS-PAGE was made and operated according to the methods in Molecular Cloning: A Laboratory Manual (3rd ed.) [18].

### 3.6. UV-Vis Spectrum Scan of TPC-A Aqueous Solution

The ultraviolet–visible (UV-Vis) spectra of TPC-A dissolved in deionized water (100 μg/mL) were scanned with a spectrophotometer (SHIMADZU UV-2550, Kyoto, Japan) in the 200–700 nm range.

### 3.7. Determination of Zeta Potentials

The Zeta potentials of TPC-A in water (1 mg/mL) were determined with a Zetasizer Nano-ZS apparatus equipped with an MPT-2 pH autotitrator (Malvern Instruments, Worcestershire, UK) that adjusted the pH of the suspension stepwise from 2.0 to 8.5 using solutions of HCl and NaOH at an interval of 0.5 and at the temperature of 25 °C [15].

### 3.8. Stability of TPC-A and EGCG Aqueous Mixtures

Three mixed aqueous solutions containing TPC-A (5 mg/mL)/EGCG (8 mg/mL), TPC-A (10 mg/mL)/EGCG (8 mg/mL), and TPC-A (15 mg/mL)/EGCG (8 mg/mL) were prepared, respectively. A mixed aqueous solution containing 1 mg/mL BSA and 8 mg/mL EGCG was used as control. The stability of these aqueous mixtures was measured by the absorbance at a wavelength of 520 nm at 25 °C, which was recorded at an interval of 2 h using a TU-1990 UV spectrophotometer (Persee General Instrument Co., Beijing, China). The experiment was conducted at 25 °C.

### 3.9. Medium-Chain Triglycerides Emulsion Stabilized with TPC-A

Medium chain triglycerides (MCT, oil phase) were blended with distilled water containing 1.0 wt. % TPC-A prepared emulsion (15.0 wt. % of oil phase, 85 wt. % of aqueous phase) by using a high-speed PT-MR2100 Polytron-type mixer (Kinematica Co., Luzern, Switzerland) at 26,000 rpm for 3 min, followed by one pass through the high-pressure homogenizer (Microfluidics M-110L, Westwood, CA, USA) at 75 MPa. To minimize possible lipid oxidation, the whole process was carried out in an ice bath. The physical stability of the emulsion was evaluated by using a storage acceleration test at 60 °C for a period of 8 days and measuring the particle size and particle size distribution. The emulsion storage test at 25 °C for a period of 8 days was regarded as a comparison. The MCT emulsion with no addition of TPC-A was prepared as a control in the same way. PSD of the emulsions was measured using a laser light scattering instrument (MasterSizer 2000, Malvern instruments Ltd., Worcestershire, UK) [19,20].

### 3.10. Moisture Absorption

Water vapor sorption properties of TPC-A were investigated for the humidity conditions of its storage, which were determined using a dynamic vapor sorption apparatus (DVS intrinsic, Surface Measurement Systems, London, UK). After pre-equilibration, 2.0–5.0 mg of TPC-A was placed onto a pre-cleaned sample pan and then carefully placed on the loop wire in the sample chamber that would be closed afterwards. The experimental conditions for water vapor sorption/desorption were pre-programmed. The temperature was controlled at 25 °C throughout, and the relative humidity was varied programmatically. When the mass of TPC-A leveled off in the starting RH condition of 0%, the RH was increased to step at 10% increments until reaching 95%, and then decreased in the same ramping rate. A dm/dt (mass variation over time variation) of 0.00002%∙min^−1^ was performed to determine the mass equilibrium at each humidity.

## 4. Conclusions

Previous studies on tea polysaccharide conjugates focused on their polysaccharide fraction. In fact, the polysaccharide conjugates were still under-explored, leading to a lack of a real understanding of its protein moiety. This status leads to the lack of reliable technical indicators and parameters in the production and quality control of tea polysaccharide conjugates. Meanwhile, high quality tea polysaccharide conjugate products are scarce in the market. Thus, this research provides two important and novel results about TPC-A. Firstly, the protein of TPC-A is enclosed within the polysaccharide chains and the physicochemical properties of the protein are shielded by the polysaccharide portion. Secondly, TPC-A has good water-holding performance and emulsifying property. Overall, this study will promote the development of applications and market prospects as well as enhance the value of TPC-A.
